# A Proof-of-Concept Preclinical Study Using a Novel Thermal Insulation Device in a Porcine Kidney Auto-Transplantation Model

**DOI:** 10.3390/ijms232213806

**Published:** 2022-11-09

**Authors:** Lisa Ernst, Zoltan Czigany, Pascal Paschenda, Mareike Schulz, Lukas Breuer, Janosch Kunczik, Michael Czaplik, Wenjia Liu, Decan Jiang, Uwe Klinge, Sonja Djudjaj, Peter Boor, Georg Lurje, Eiji Kobayashi, René H. Tolba

**Affiliations:** 1 Institute for Laboratory Animal Science and Experimental Surgery, Faculty of Medicine, RWTH Aachen University, 52070 Aachen, Germany; 2 Department of Surgery and Transplantation, University Hospital RWTH Aachen, 52070 Aachen, Germany; 3 Department of Surgery, Campus Charité Mitte/Campus Virchow Klinikum, Charité Universitätsmedizin, 13353 Berlin, Germany; 4 Department of Anesthesiology, Faculty of Medicine, RWTH Aachen University, 52070 Aachen, Germany; 5 Institute of Pathology, University Hospital RWTH Aachen, 52070 Aachen, Germany; 6 Department of Kidney Regenerative Medicine, The Jikei University School of Medicine, Tokyo 105-8461, Japan

**Keywords:** kidney transplantation, organ insulation, ischemic reperfusion injury

## Abstract

Ischemia-reperfusion injury remains a fundamental problem during organ transplantation logistics. One key technical factor is the rapid allograft rewarming during the time of vascular reconstruction in the recipient. In this pilot study, a new thermal insulation bag (TIB) for organ transplantation was used. Insulation capacity, tissue compatibility, and usability were tested initially ex vivo on porcine kidneys (n = 24) followed by the first in vivo usage. Fourteen female German landrace pigs underwent kidney auto-transplantation after 24 h cold storage (4 °C). During the implantation process the kidney was either insulated with the new TIB, or it was not thermo-protected at all, which represents the clinical standard. In this proof-of-concept study, the usability (knife-to-skin-time) and the general thermal capacity (30 min warm storage at 38 °C ex vivo *p* < 0.001) was shown. The clinical outcome showed significant differences in the determination of CRP and pi-GST levels. Syndecan-1 Antibody staining showed clear significant higher counts in the control group (*p* < 0.01) indicating epithelial damage. However, the effect on renal outcomes in not severely pre-damaged kidneys does not appear to be conclusively significant. A close follow-up study is warranted, especially in the context of marginal organs or in cases where anastomosis-times are prolonged due to surgical complexity (e.g., multiple vessels and complex reconstructions).

## 1. Introduction

Kidney transplantation still represents the only curative treatment for end-stage renal failure that provides the best quality of life and reduced long-term morbidity compared to dialysis. Due to demographic changes of the past decades, a decreasing quality of the donor pool (e.g., elderly donors with comorbidities) is combined with an ever-increasing organ shortage and soaring numbers of patients with end-stage renal disease.

In Europe, only between 20% [1] and 25% [2] of patients on the waiting list can receive a live-saving suitable donor organ each year, leading to a high waiting list morbidity and mortality [3].

A major factor influencing the quality and viability of the graft is the so-called ischemia-reperfusion injury (IRI). This process is mainly divided into the phase of warm and cold ischemia and the phase of reperfusion [4]. The phase of warm ischemia consists of 2 distinct insults: (I) The stage of organ procurement; (II) The time required in situ for implanting the kidney into the recipient (second warm ischemic time (SWIT)) [5].

In a clinical setting, kidneys are stored cold at 4 °C in a static cold storage solution until transplantation [6,7]. Static cold storage aims to slow down the metabolic processes and to minimize the oxygen demand of the kidney [7]. This method has been the clinical gold standard in the field of kidney transplantation since the pioneering years of solid organ transplantation [8]. However, a problem that has not yet been addressed and arises during implantation of the kidney into the recipient, is the SWIT. During the anastomosis of the renal artery and renal vein, the graft is already exposed to body temperature without being supplied with blood and oxygen. Warm ischemia (WI) causes tissue damage that may contribute to subsequent delayed graft function or even primary non-function [9,10,11]. To reduce or avoid this WI, cooling devices are used experimentally. Among these approaches, the “ice bag technique” can be used [12,13]. Here, the graft will be placed in an ice-filled bag with an outlet for the renal vessels. However, this technique is often criticized as it carries the risk of further damage by freeze burn [13]. Alternatively, cold temperatures during implantation can be achieved by either using a cooling device or insulating jackets. This approach was established as the global trend in recent years for development of medical devices to address this issue [14,15,16,17,18]. A thermal insulation bag (TIB) with innovative design would be clinically attractive and holds promise to replace the simple “ice bag”. This proof-of-concept study shows the results of the ex vivo testing and the first in vivo usage of a new TIB in a porcine kidney auto-transplantation model.

## 2. Results

### 2.1. Ex Vivo—Testing

**Figure 1:**
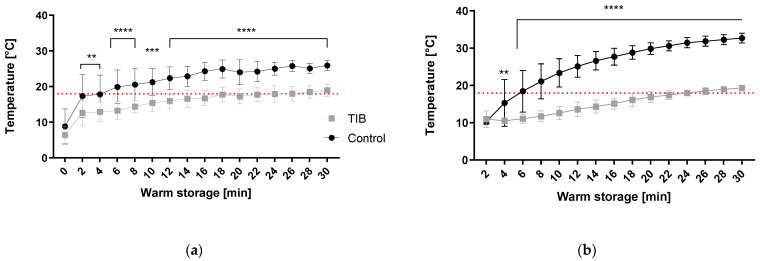
Temperature increase in a water bath at 38 °C for 30 min. (**a**) Measurement of the superficial temperature (outside) (**b**) Measurement of the core temperature (inside). Red dashed line shows 18 °C threshold for WI. ** *p* < 0.01, *** *p* < 0.001, **** *p* < 0.0001.

Figure 1 illustrates the results of ex vivo testing of the thermal insulation capacity of the TIB on the surface (a) and core temperature (b). The results show highly significant differences in the development of both surface and core temperature during the measurement phase of 30 min. While the increase gradient of the surface temperature is very similar in the graph, the increase in the core temperature in the TIB group is slowed down significantly. This is displayed in Figure 1b by a clearly flatter curve in the TIB, which leads to a highly significant difference (adjusted *p* value < 0.0001) in the core temperature values at the end of the measurement.

### 2.2. In Vivo Testing

#### 2.2.1. Applicability, Surgical Management

**Figure 2:**
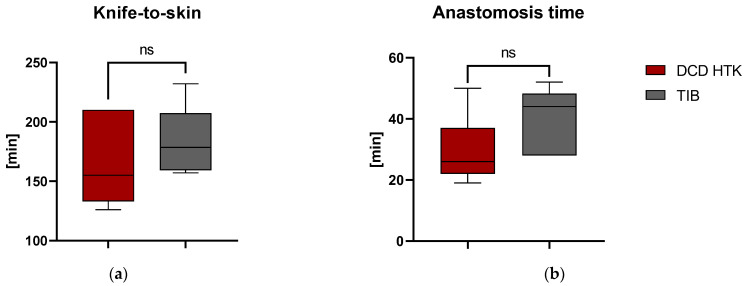
Duration of surgical intervention (**a**) Duration of surgery in knife-to-skin time (**b**) Duration of anastomosis time before reperfusion. Values are reported with their respective median and 95% confidence interval. DCD = Donor after cardiac death.

The effect of using the TIB for surgeries and the general applicability of the TIB was investigated by measuring the duration of surgical intervention. Duration of surgery (Figure 2a) was defined by determining the knife-to-skin time and showed non-significant differences between the groups. Similarly, time from initiation of anastomosis to reperfusion was 34 min (SD = 10.45 min.) and showed no significant differences (*p* > 0.089), although there might be a slightly higher median value indicating a trend towards a longer suturing time in the TIB group shown in Figure 2b.

#### 2.2.2. Serum Values, Clinical Outcome

**Figure 3:**
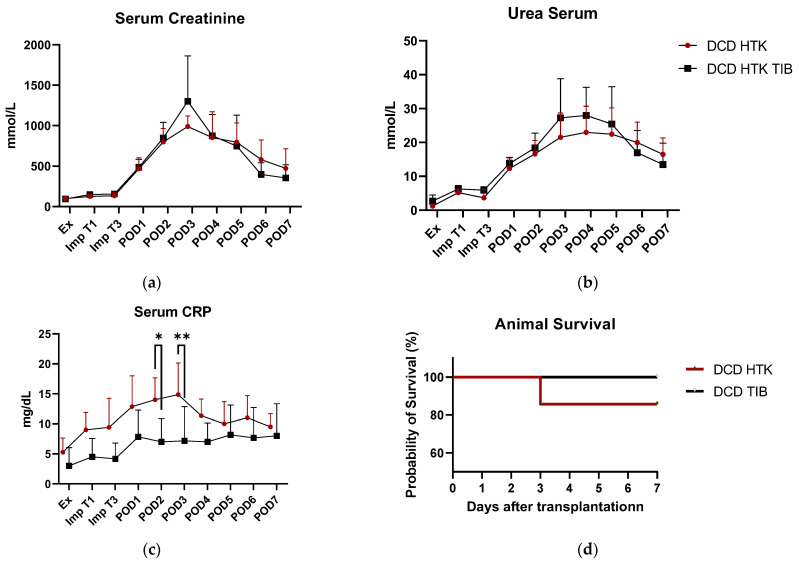
Exemplary results of clinical values and survival for 7 days post operatively = POD. (**a**) Creatinine serum levels (**b**) Urea serum levels (**c**) CRP Serum levels (**d**) Animal survival. * *p* < 0.05, ** *p* > 0.01 2-Way ANOVA Sidak’s multiple comparison.

Figure 3a–c shows exemplary serum levels of creatinine (a), urea (b), and CRP-values (c) for the analysis of clinical status of renal function. As an indication of early graft failure, survival of animals during the observation period is displayed in Figure 3d. Both urea and creatinine showed a clinically relevant increase in the first 3–4 days after renal transplantation but showed no significant differences between the groups. However, there was also no significant difference in other clinical values such as urine output, creatinine clearance, potassium values, or renal protein excretion (Supplemental Material Figures S1–S4). One of the animals in the HTK control group reached humane endpoints due to primary non-function after POD3 (Figure 3d).

#### 2.2.3. Thermographic Imaging

**Figure 4:**
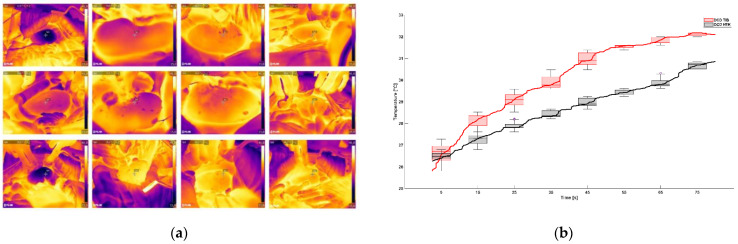
Thermographic Imaging. (**a**) Exemplarily illustration of the thermal camera imaging during reperfusion. (**b**) The graph shows the determination of surface temperature over time of 80 s. Red line corresponds to the HTK control group, black line to the TIB group. *p* adjusted *p* = 0.76, 2-Way ANOVA Kruskal-Wallis test.

Thermographic video signals were able to quantify the rewarming of the renal surface over time in the period right after reperfusion and are displayed in Figure 4a,b. Average starting temperature at the beginning of reperfusion was 25.17 °C (SD = 1.87) for the TIB and 25.64 °C (SD = 1.57), respectively. The average graph per group shows no significant difference in temperature decrease with a *p* value of *p* = 0.76.

#### 2.2.4. Histopathological Evaluation

**Figure 5:**
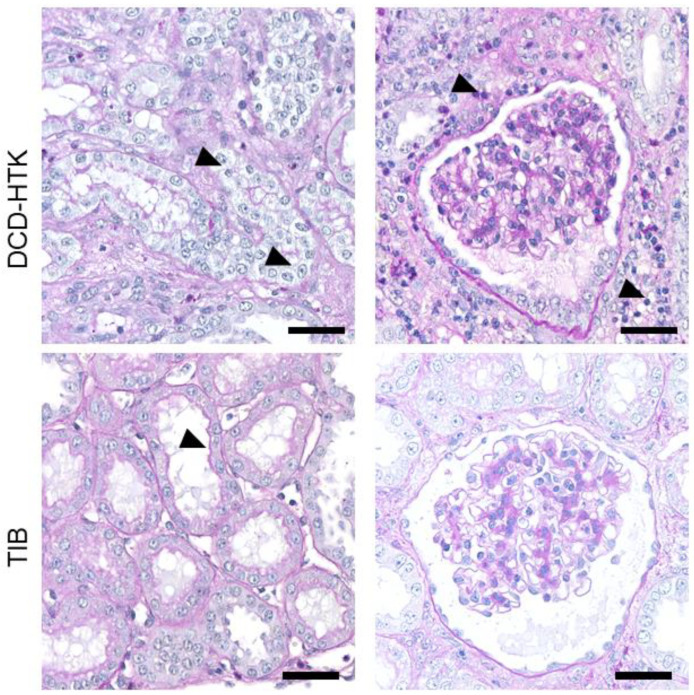
Histological appearance of kidneys 7 days after transplantation in PAS staining in the DCD HTK (**above**) and HTK TIB group (**below**). Black arrows show tubular damage (loss of villi and edema, respectively) in column 1, in column 2 marking of immune cells. Magnification 200×, Scale bars 20 µm.

Following the study, semiquantitative histological analysis was performed in HE sections using renal injury scores to determine acute kidney injury (AKI). The score includes the following criteria: infiltration of immune cells (neutrophil granulocytes and lymphocytes); damage to tubular cells (loss of brush border, dilatation, atrophy, and necrosis), deposition of extracellular matrix (fibrosis) and edema. The classification is as follows, depending on the extent: Score 0 = no damage up to score 5 = diffuse damage and shown in Table 1. Exemplary sections of HTK and TIB in PAS staining (Figure 5) show low to moderate damage and correspond to the findings in Table 1. PAS staining shows no endothelial damage neither in glomerular nor tubular areas.

**Figure 6:**
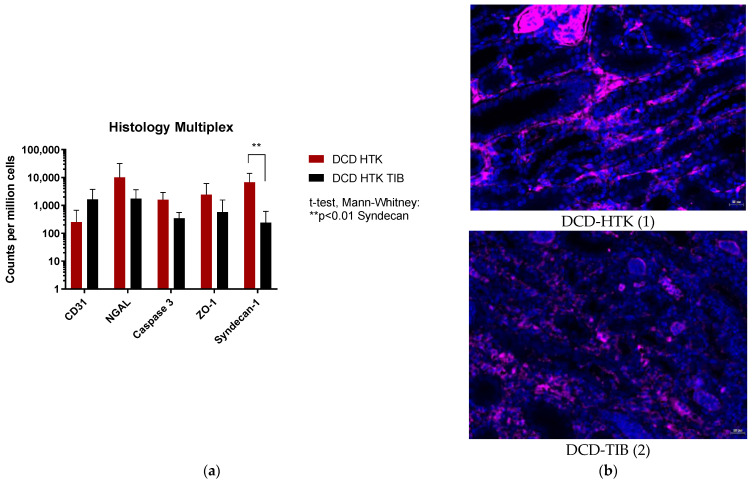
(**a**) Histological Evaluation with Multiplex antibody staining for CD31, NGAL, Caspase 3, ZO-1, and Syndecan-1 positive cells. Mann–Whitney test *p* adjusted= 0.0012 (**b**) Appearance of syndecan-1 positive cells (pink fluorescence) with DAPI nuclear staining in the DCD HTK (1) and HTK TIB group (2). Magnification 200×, Scale bars 20 µm. Images are created with TissueFAXS Viewer 7.1.120.

Histological evaluation of kidney damage was performed by counting antibody positive cells for CD31, NGAL, Caspase 3, ZO-1 (membrane protein zona occludence-1), and Syndecan-1 with multiplex staining. Analysis of Syndecan-1 showed significant differences (*p* < 0.01; *p* adjusted = 0.0012) between the DCD–HTK and the TIB group. No significant differences are detected in CD31 (*p* adj = 0.051), NGAL (*p* adj = 0.53), Caspase3 (*p* adj = 0.101), and ZO-1 (*p* adj > 0.99). Figure 6 shows an exemplary appearance of syndecan-1 positive staining in both groups; other color filters of the multiplex were switched off for better overview.

#### 2.2.5. ELISA

ELISA assays in serum (NGAL; TNF-alpha, pi-GST) and urine (KIM-1, normalized to creatinine levels) also show low to moderate levels of acute kidney injury during the first 3 days after surgery. Whereas NGAL and TNF-alpha did not show any significant differences, a significant reduction of pi-GST values was observed at POD 1 in the TIB group. KIM-1 values showed a significant difference on POD1 towards a higher level in the TIB group. Despite a very high variance, no outliers could be detected.

## 3. Discussion

The aim of this proof-of-concept study was the in vitro and in vivo evaluation of a new thermal insulation device for kidney transplantation. SWIT is a key player in the extent of IRI. The effect of SWIT on IRI strongly depends on the temperature and the time when this temperature is exceeded. Therefore, minimizing damage in SWIT is of utmost importance for post-transplant graft functionality. One approach to this is to minimize the temperature to keep the SWIT-induced damage low. It could be shown in literature that the critical degree for resumption of hypoxic metabolic performance is based on a temperature of 15 °C [5,19] leading to warm ischemia. A temperature of >18 °C is considered critical in terms of tissue injury and can be reached approx. 20 min after termination of the cooling phase at the backtable [20]. In our preceding ex vivo study, this cutoff (Figure 1a,b red dashed line) of 18 °C is already reached at 2 min (outside) or 6 min (inside) in unsheathed kidneys. In the TIB group, this critical line is not exceeded until 18 min (outside) or 24 min (inside). Thus, a significantly reduced slope of the core temperature increase can be achieved, demonstrating the insulation capacity of the test device. In the past 5 years, several investigations targeted the insulation and cooling capacities of various devices during kidney transplantation. In the study by Torai et al., thermal insulation was achieved by using a thermal barrier bag (TBB) consisting of a two layer system including a thermal barrier sheet used in a porcine kidney transplantation model [15]. The TBB is an insulation device made of medical grade silicone [18] and can be seen as a preliminary version of the device shown here with a more rigid and less elastic surface. Likewise, the ex vivo approach of insulation performance in a 37 °C water bath was investigated first, with results reaching >18 °C threshold approx. at 14 min and 30 °C after 30 min on the surface of the kidney. In our results presented here, 30 °C was never reached within the 30 min observation period (Figure 1a,b). However, a critical temperature of over 18 °C had been reached already after approx. 3 min in the control group, which is in line with our control results shown in Figure 1a.

Khan et al. arrived at slightly different conclusions in 2021, using different insulation ‘jackets’ based on polyurethane and silicone [14]. While the water bath temperature was also set to a comparable level of 37 °C, they only measured core temperature. These measurements resulted in reaching the 15 °C mark at approx. 17 min and the 18 °C threshold at approx. 21 min in control kidneys. In our study, (Figure 1b) an increase in core temperature already reached 15 °C level after 4 min, and 18 °C after 6 min in the control group, respectively. Khan et al. achieved significant extensions from 21 min in the control group up to 38 and 49 min to the critical temperature in their respective experimental groups. Thereby insulation capacity was thus able to double the warming time. In our study, the heating time could be extended from 6 to 22 min (Figure 1b), resulting in a 3-fold increase in the critical time span. However, measurements in core temperature were not feasible during in vivo investigations due to their invasive nature, potential damage to kidney tissue, and the risk of post-operative bleeding.

The comparable designs of the mentioned ex vivo studies allow the conclusion that the use of TIB has similar general thermal insulation properties as the use of polyurethane and silicone as described by Khan et al. or Torai et al.

General applicability could be demonstrated, as no significant differences between surgery (Figure 2a) and anastomosis times (Figure 2b) resulted from the use of the device. However, median values in the TIB group showed a slight trend towards higher values. This might be attributed to a certain unfamiliarity with the device or a certain “learning curve” of the respective surgeons, even though this effect seemed negligible.

**Figure 7:**
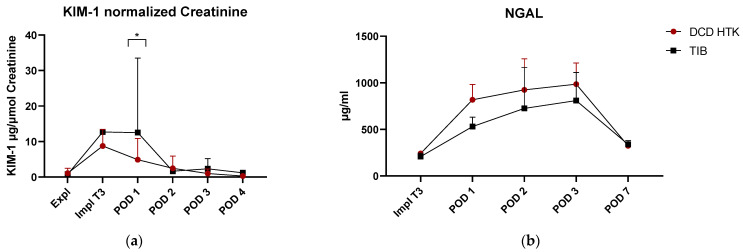

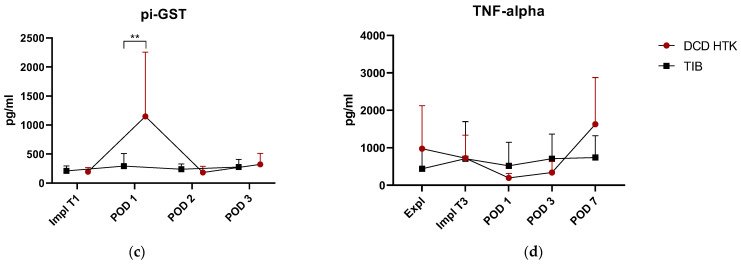
Results of ELISA measurements. (**a**) KIM-1 normalized to Creatinine Values * *p* < 0.05 (n = 4–6) (**b**) NGAL = Neutrophil Gelatinase Associated Lipocalin (n = 2–6). (**c**) pi-GST ** *p* < 0.01 (n = 3–6) (**d**) TNF-alpha values are displayed in boxplots with its median and 95% confidence intervals (n = 5–6) ** *p* < 0.01 2-Way ANOVA Sidak’s multiple comparison.

With regard to clinical results, no significant differences could be detected in the values of serum creatinine (Figure 3a), Urea (Figure 3b), and other parameters (urine release, creatinine clearance, potassium values, or renal protein excretion (Supplementary Material Figures S1–S4)). The progression of the creatinine increase, with peak values between day 2 and day 3 postoperatively as well as their absolute values are in compliance with the TBB study results and also underline the similarity of the respective parameters. CRP values differed significantly on POD 2 and 3 (Figure 3c.). However, TNF-alpha values (Figure 7d) showed no decisive results, leading to the conclusion that CRP values in this study are not triggered by infection. One animal of the control group reached humane endpoints (Figure 3d) and was sacrificed, retrospectively caused by non-function of the kidney, which could be indicative of the protective effect of TIB use.

Temperature development at the start of reperfusion by thermographic analysis in the first 80 s showed no significant differences between the groups. Onset temperature was 25.17 °C (SD = 1.87) for the TIB and 25.8 °C (SD = 1.57) for the control group and was achieved approx. 29 min in the control and 40.5 min for the TIB group after the start of anastomosis (Figure 2b). This can be used to explain the lack of significance in the onset temperature. The average length of surgery for kidney transplantation is usually considered to be 35 min; longer surgery times are associated with surgical difficulties or challenges [5,21]. In our study, prolonged operation times >45 min occurred twice in the TIB group and none in the control group. This must therefore be taken into account when interpreting the thermographic results. This can also be considered in comparison to the use of the TBB as a comparative device, likewise reporting a temperature of about 24 °C after 35 min [15]. Semi quantitative histopathological analysis of scores of renal tissue injury shown in Table 1 did not detect any significant differences in the use of TIB and the control group. This is shown by exemplary images of histological sections in PAS staining for the control group and TIB (Figure 5). Similar results were also shown in the use of an intra-abdominal cooling device [22]. In the 2019 study by Longchamp et al., a cooling device in the porcine model of kidney transplantation was also used, achieved by continuous circulation of ethanol and methylene blue at a temperature of 4 °C. [17]. Despite promising results in some data, no clear significance could be shown in the context of tissue injury. Overall, the degree of histological damage in the study shown here is classified as mild to moderate (Table 1). This is also underlined by the results of ELISA analysis (Figure 7a–d). While pi-GST (Figure 7c) as a marker for tubular cell damage shows significant differences as a single index, NGAL, and TNF-alpha did not show any significant between-group differences and their value rather corresponded to moderate renal damage. Results of KIM-1 showed a significant difference on POD1 which could not be fully explained and maybe was caused by the relatively low sample size and high deviation. However, despite the lack of significant differences, the median values of the TIB group were below those of the control group in the majority of investigations.

Analysis of multiplex staining (Figure 6a) shows a significantly higher number of syndecan-1 positive cells in the control group. High values of syndecan-1 (Figure 6b) indicate degradation of endothelial glycocalyx associated with endothelial damage and acute renal injury [23]. This result may underline the findings in significantly different pi-GST values revealing a clinical effect of the TIB which might have been more prominent in a larger cohort with more statistical power.

## 4. Materials and Methods

### 4.1. Ethical Statement

The study was designed and executed in accordance with the EU Directive 2010/63 and the Guide for the Care and Use of Laboratory Animals [24]. The study was performed and reported in accordance with the ARRIVE guidelines [25] (Supplemental Material File S1). All surgical procedures have been performed under general anesthesia. Sufficient analgesia was ensured using buprenorphine (Temgesic, Essex pharma, Munich, Germany, 8 mg/kg Bodyweight) three times daily for the first 72 h. Study protocol was approved by Governmental Animal Care and Use Committee of the federal state of North Rhine-Westphalia (Ladesamt für Natur- Umwelt- und Verbraucherschutz (LANUV) Recklinghausen, Germany) under License No. 02.04.2018.A051.

### 4.2. Experimental Study Design

The ex vivo tests were performed using a temperature-regulated water bath for testing the thermal insulation capacity of the TIB. The kidneys for ex vivo analyses were retrieved from 12 female German landrace pigs directly after euthanasia by overdose of pentobarbital (60 mg/kg) without further treatment. Following the 3R principle, other porcine organs and blood have been made available for other studies. After kidney retrieval, the artery was cannulated and flushed with 250 mL saline followed by preservation with 250 mL HTK solution and cold storage at 4 °C for 100 min. The kidneys then were successively warmed either with or without the TIB floating in a water bath at 40 °C water temperature for 30 min.

A Power calculation with the use of the G-Power Software [26] (G-Power, Version 3.1.9.4, Heinrich Heine University, Düsseldorf, Germany) was performed prior to the experiments for sample size estimation. Animals were randomly assigned to their respective groups.

**Figure 8:**
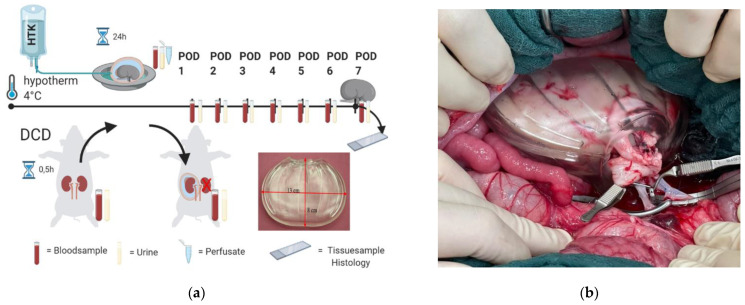
(**a**) Overview of the study design of the in vivo experiment. Schematic illustration was created with BioRender.com TIB prototype used as the test product as a high elastic, bag-shaped device for organ protection and thermal insulation (**b**) TIB placed in situ during anastomosis.

For the in vivo study, a total of 14 female German landrace pigs of 37.9 kg (SD = 4.5 kg) bodyweight were used. One animal from the HTK-group had to be finalized during surgery and was therefore excluded from the study. All animals received a 14-day acclimatization period before entering the study. Animals were group-housed with 12 h day night cycle (7:00 a.m./7:00 p.m.) before the study and single housed during the postoperative phase of the study with the possibility of visual and olfactory contact to their companions. Feeding was performed 2 times a day with standardized pellets (Alleinfuttermittel für Mastschweine, sniff Spezialdiäten GmbH, Soest, Germany). Water was measured and replaced, 5 L each morning and evening. Kidney transplantation was performed under general anaesthesia following our published protocol for kidney auto-transplantation in pigs [27]. The design of the study is shown in Figure 8a. For organ retrieval, the abdomen was opened via a midline incision of approx. 25 cm, followed by exposure of the left kidney after opening the retro-peritoneum. To mimic the Donation after Cardiac death (DCD) scenario, blood flow in the left kidney was interrupted by clamping the renal artery and vein for 30 min before retrieval. No heparin was administered in this phase. For flush out of the kidney graft, the renal artery was cannulated with a 16 GA (gauge) vein cannula (Becton, Dickinson and Company, Franklin Lakes, NJ, USA) on ice. The flush out was performed with 200 mL ice cold 0.9% saline (B. Braun Melsungen AG, Melsungen, Germany) with 1.200 IU heparin (B. Braun Melsungen AG, Melsungen, Germany). Thereafter, the kidney graft was perfused with 500 mL Bretschneider’s Histidine, Tryptophan, and α-Ketoglutarate solution (HTK) (Custodiol, Dr. Franz Köhler Chemie GmbH, Bensheim, Germany) at 4 °C. The flush out and perfusion were performed with a perfusion pressure of 100 cm water column. After the flush out, the kidney was weighed and stored in HTK at 4 °C for 24 h.

After the 24-h preservation period, the abdominal cavity was re-opened and the contralateral kidney was removed, followed directly by orthotopic auto-transplantation of the left kidney. In the DCD HTK TIB group, the kidney was stored in the TIB (Figure 1B) during time of anastomosis; in the DCD HTK group (control group) the kidney was disposed on a saline wet cotton gauze. During the first 100 s of reperfusion, re-heating of the kidney was filmed using an infrared thermal imaging camera System (Model FLIR 95, Teledyne FLIR, Wilsonville, OR, USA). The videos were evaluated in MATLAB (The MathWorks Inc., Natick, MA, USA) by importing them using FLIR’s MATLAB SDK. The kidney outlines were manually selected as Region of Interest (RoI) using the imfreehand-Tool. The RoI’s mean temperature was computed for every frame. Occlusions, or movements of the RoI were manually corrected on a frame-by-frame basis by excluding disturbed frames and by reselection of the RoI after disturbances. Missing mean temperatures due to disturbed frames were computed by means of linear interpolation.

After 7 days post transplantation or if the animal reached the humane endpoint criteria according to our score sheet, the animals were sacrificed in anesthesia with an overdose of pentobarbital (Narcoren, Boehringer Ingelheim, Germany, Dosage of 60 mg/kg BW).

### 4.3. Histology

Kidney tissue was collected and fixed using 4% formaldehyde solution for 48 h. Following dehydration, the kidney tissue was embedded in kerosene and appropriate sections were fabricated. Histological evaluation was performed by staining with HE and PAS. Subsequently, a semi quantitative histological analysis was performed using a renal injury score to determine acute kidney injury (AKI). The score includes the following criteria: infiltration of immune cells (neutrophilic granulocytes and lymphocytes), damage to tubule cells (loss of brush border, dilatation, atrophy, and necrosis), deposition of extracellular matrix (fibrosis), and edema. The classification was conducted according to the following levels: Score 0 = no damage, score 1 = 0–10%; score 2 = 10–25%; score 3 = 26–50%; score 4 = 51–75%; Score 5 = 76–100%. The assessment was performed in a blinded process.

Immune histology was performed with immunofluorescence staining analogously to a previously published protocol [28]. A 5-fold panel was performed to screen the kidney. The markers analyzed in the panel were CD31 (cell death), NGAL (acute kidney injury), caspase 3 (apoptosis), ZO-1 (membrane protein zona occludence-1), and Syndecan-1 (endothelial damage). Images were quantitatively analyzed using StrataQuest Analysis Software (v7., TissueGnostics, Vienna, Austria). In each case, a whole section was used for the examination, which included both the cortex and the medullary region of the kidney. A mean intensity > 100 cells was considered to be positive. The results were normalized to 1,000,000 cells to ensure comparability.

### 4.4. Data Analysis

Statistical analysis and data evaluation were performed using GraphPad Prism [29] Version 9.2.0. A 2-Way ANOVA with mixed effect model was chosen with Sidak’s multiple comparison (Temperature, Creatinine, Urea, CRP values, ELISA) due to missing values and One-Way ANOVA; for results of thermal imaging Kruskal-Wallis multiple comparison was used too. A t-test was performed for nonconsecutive measurements along group comparisons (duration, histology). All values are proved for normality in advance. Values are given in mean and standard deviation except if described otherwise. Results were considered as statistically significant if adjusted *p*-value was <0.05.

### 4.5. Thermal Insulation Bag

The thermal insulation bag is a high elastic, bag-shaped device (Figure 7b) for organ protection and thermal insulation made of uniquely developed low-hardness styrenic elastomer gel (Patent No.JP2020044287A/JP2021126384A/JP2021040938A, Kawasaki, Japan). The TIB is an elastomer gel molded product with an elongation rate of approx. 1000% and a melting point of more than 200 °C, biologically safety tested and with a Shore A0 hardness scale.

## 5. Conclusions

In summary, this study demonstrates the applicability of TIB as a new tool in a preclinical investigation to minimize SWIT and IRI. The ability for temperature insulation could be shown ex vivo with clear evidence and is comparable to similar projects or improved. No graft loss compared to the control group could be observed. In some parameters, a trend for improvement of the clinical and metabolic parameters can be seen. The lack of significant between-group differences can be explained mainly by the low to moderate damage also in the control group. The effect, which is clearly visible ex vivo, can only be detected in the clinical application with a high number of animals due to a too small difference (delta). The first evidence of significant clinical protection at the molecular level is accessible by decrease in tubular damage shown in lower syndecan-1 expression. In order to increase the power to investigate the protective effect, further subsequent studies are therefore necessary. This should be investigated in further studies including higher degrees of injury.

## Figures and Tables

**Table 1:** Histological Evaluation of HE stained kidney sections, n = 7.

Group	Inflammation & Infiltration	Hemorrhage	Glomeruli Damage	Tubular Damage	Edema
DCD HTK	2.9 ± 0.6	2.3 ± 0.9	2.2 ± 0.4	2.5 ± 0.6	2.4 ± 1.1
DCD HTK TIB	3.2 ± 1.2	2.8 ± 1.5	2.5 ± 0.6	2.7 ± 0.7	2.9 ± 0.8

## Data Availability

The data that support the findings of this study are available stored online in zenodo respository at URL https://doi.org/10.5281/zenodo.7034746 accessed 7 August 2022. Further enquiries can be directed to the corresponding author.

## Supplementary Materials

The following supporting information can be downloaded at: https://www.mdpi.com/article/10.3390/ijms232213806/s1, Figures S1–S4 (urine output, creatinine clearance, potassium values or renal protein excretion). File S1. The ARRIVE Guidelines Checklist. References [30,31] are cited in Supplementary Materials.
