# Supplementary material for: A Proof-of-Concept Preclinical Study Using a Novel Thermal Insulation Device in a Porcine Kidney Auto-Transplantation Model
Source: Int J Mol Sci. 2022 Nov 9;23(22):13806. doi: 10.3390/ijms232213806 (PMC9697795; doi:10.3390/ijms232213806)
Supplement: Supplementary file 1 [file ijms-23-13806-s001.zip › ijms-1919794-supplementary.pdf]

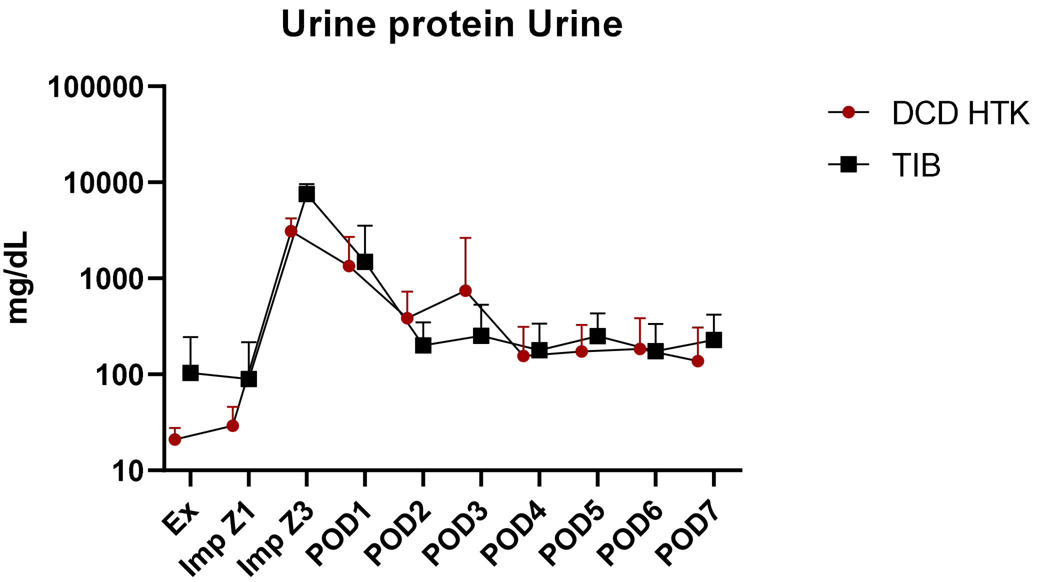

Figure S1. Protein values of Urine in mg/dl over 7 days after surgery. No significant differences can be shown between the group DCD HTK and TIB.

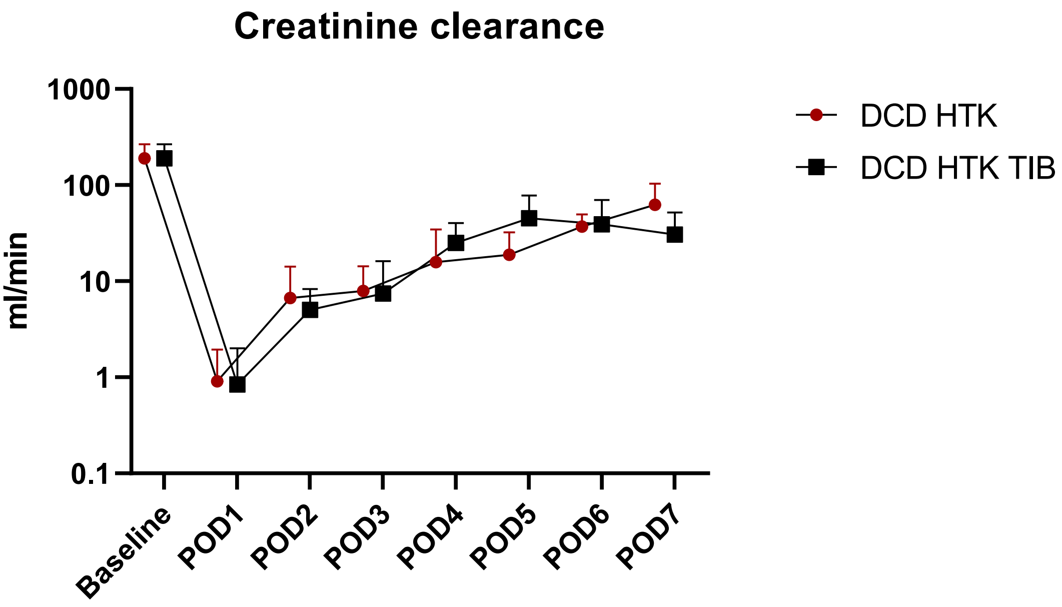

Figure S2. Creatinine clearance over 7 days after surgery. No significant differences can be shown between the group DCD HTK and TIB.

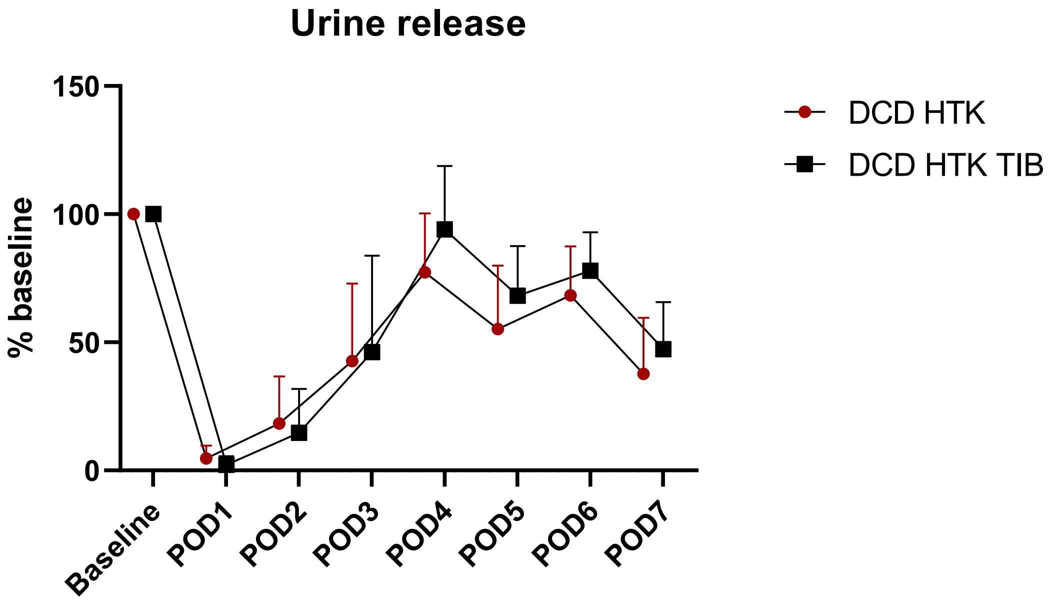

Figure S3. Urine release normalized to individual Baseline over 7 days after surgery. No significant differences can be shown between the group DCD HTK and TIB.

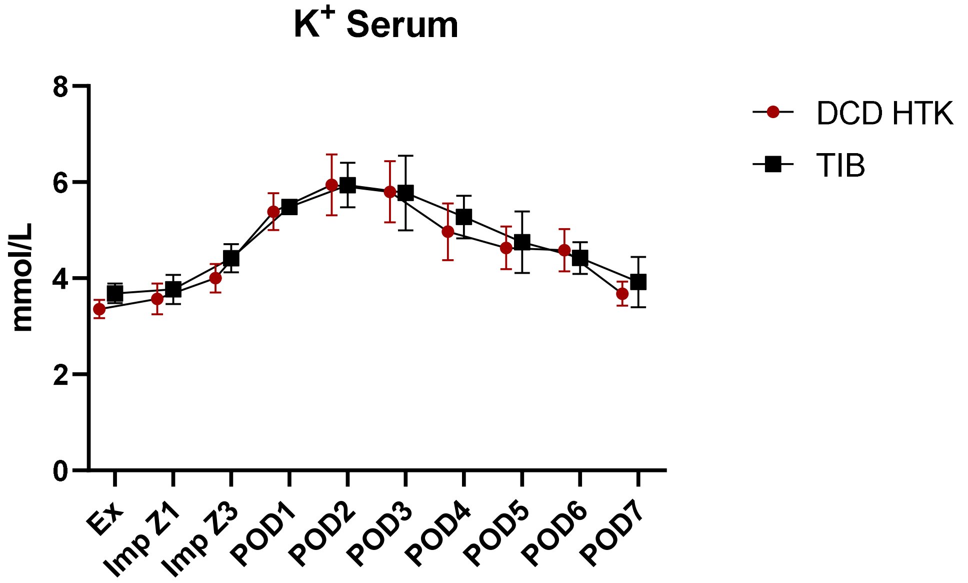

Figure S4. Potassium serum level over 7 days after surgery. No significant differences can be shown between the group DCD HTK and TIB.
